# Current Trends of Wide-Awake Hand Surgery in the United States

**DOI:** 10.3390/jcm15145446

**Published:** 2026-07-11

**Authors:** Alexander J. Kammien, Andrew Salib, Adnan Prsic, Jonathan N. Grauer, David L. Colen

**Affiliations:** 1 Division of Plastic Surgery, Department of Surgery, Yale School of Medicine, New Haven, CT 06510, USA; alexander.kammien@yale.edu (A.J.K.); andrew.salib@yale.edu (A.S.); adnan.prsic@yale.edu (A.P.); 2 Department of Orthopaedics and Rehabilitation, Yale School of Medicine, New Haven, CT 06510, USA; jonathan.grauer@yale.edu

**Keywords:** WALANT, office-based surgery, outpatient, hand surgery, cost, reimbursement, wide-awake, carpal tunnel, trigger finger

## Abstract

**Background/Objectives**: This national database study compares wide-awake hand surgeries in the United States performed in the operating room and office in terms of volume, reimbursement, narcotics prescriptions, and adverse events. **Methods**: Patients who underwent trigger finger release, open carpal tunnel release, De Quervain’s release, and mucous cyst excision from 2010 to 2022 were identified in PearlDiver’s M170Ortho dataset. Exclusion criteria were concomitant hand surgery, inpatient setting, <30 days of follow-up, age < 18 years, general/monitored anesthesia, and nerve block. Cohorts were stratified by surgical setting then matched by age, sex, Elixhauser Comorbidity Index score, and region. Primary endpoints included total and physician reimbursement (stratified by payor: commercial, Medicaid, Medicare) and 90-day narcotic prescriptions, emergency department visits, and surgical site infections. **Results**: Between 2010 and 2022, all surgical cohorts demonstrated an increase in the annual proportion of surgeries performed in the office (trigger finger +36%, carpal tunnel +155%, De Quervain’s +104%, mucous cyst +22%). Office-based surgery demonstrated lower total costs for all surgical cohorts and insurance types: commercial (−34% to −43%), Medicaid (−37% to −48%), Medicare (−30% to −37%). Office-based surgery had lower physician reimbursement for all surgical cohorts with commercial insurance (−3% to −9%) and Medicare (−5% to −13%). Physician reimbursement was not significantly different by surgical setting for Medicaid patients. Following office-based surgery, patients filled fewer narcotic prescriptions and had lower rates of emergency department visits, with similar rates of surgical site infection. **Conclusions**: Although most wide-awake hand surgeries are still performed in the operating room, there is a nationwide increase in office-based surgery. With reduced financial burden and favorable rates of adverse events, office-based wide-awake hand surgery may offer improved economic value and comparable short-term safety for certain procedures. Future research should complement these findings with patient-reported outcomes.

## 1. Introduction

Due to its proven safety and patient satisfaction, office-based hand surgery has become increasingly popular, especially with the development of tourniquet-free techniques [1]. The increased prevalence of the wide-awake, local anesthesia no-tourniquet (WALANT) technique has improved safety and efficiency by eliminating the sedation and cardiorespiratory monitoring typically required when using tourniquets and monitored anesthesia care [1,2,3]. Performing surgery in the office setting increases logistical and financial efficiency for patients and providers in several ways, including lower costs and administrative overhead, shorter cases, simpler scheduling, and increased operating room (OR) availability for other cases [3,4,5,6,7,8].

In a large, nationwide study, Billig et al. recently demonstrated that performing hand surgeries in the office reduces cost without increasing adverse events, but the study did not exclusively examine wide-awake surgery, making it difficult to determine the contribution of anesthesia and surgical setting to cost reductions [9]. Other authors have demonstrated the reduced cost and improved efficiency of office-based wide-awake hand surgeries compared to those performed in the OR using small single center cohorts [8,10,11]. We have also contributed several national cohort studies examining wide-awake surgery for individual procedures, including carpal tunnel release [12,13], Dupuytren fasciectomy [14], and flexor tendon repair [15]. However, the results should be further explored across multiple procedures in order to generalize and update these findings.

The current study seeks to expand on prior studies by comparing wide-awake hand surgeries in the office and OR using United States national administrative data from 2010 to 2022. Wide-awake trigger finger release, carpal tunnel release, De Quervain’s release, and mucous cyst excision were compared to determine differences in volume, reimbursement, narcotic prescriptions, emergency department visits, and surgical site infections by surgical setting. We hypothesized that, compared to surgeries in the OR, office-based surgery would become more common over time and have reduced financial burden and similar adverse events.

## 2. Materials and Methods

Database and Patient Selection. The current study analyzed data from PearlDiver’s 2010-Q1 2023 M170Ortho dataset. PearlDiver is a commercially available insurance claims database that includes data from commercial and government sources including ICD and CPT codes, demographic, reimbursement, and prescription data. The M170Ortho dataset includes information from about 171 million patients from all regions of the United States between January 2010 and December 2022. PearlDiver studies have been granted an exemption from the Institutional Review Board because all outputs are deidentified and aggregated.

Surgical cohorts were identified using CPT codes: trigger finger release (26,055), open carpal tunnel release (64,721), De Quervain’s release (25,000), and mucous cyst excision (26,160). Exclusion criteria included age < 18 years, concomitant hand surgery, <30 days of postoperative database follow-up, CPT codes for nerve blocks, sedation, monitored anesthesia care, or general anesthesia for upper extremity surgery, and inpatient surgery. Patients were stratified by surgical setting: outpatient (hospital-based outpatient departments and ambulatory surgery centers) or office, then matched 1:4 (office:OR) by age, sex, Elixhauser Comorbidity Index (ECI) score [16], and U.S. Census region using exact matching. Because the database does not directly capture use of epinephrine, absence of a tourniquet, or other technical elements that define the WALANT technique, wide-awake cases were identified indirectly by the absence of these anesthesia-related codes rather than by direct confirmation of the WALANT-specific technique. This limitation is addressed further in the Discussion.

Outcomes. Total and physician reimbursement on the day of surgery were stratified by insurance type (commercial, Medicaid, Medicare). Postoperative narcotic utilization, emergency department (ED) visits, and surgical site infections (SSIs) were assessed at 90 days postoperatively, among patients with at least 90 days of database follow-up available. ED visits were identified using CPT codes 99281–99285. SSIs were identified using ICD-9/ICD-10 diagnostic codes and CPT codes (ICD-9-D-99851, ICD-9-D-99859, ICD-10-D-T814XXA, ICD-10-D-T8140XA–T8149XA, ICD-10-D-T814XXD/S; CPT-10060, -10061, -10160, -10140, -25028).

Statistical Analysis. Statistical analyses were performed within PearlDiver with significance defined as *p* < 0.05. Chi-squared tests compared proportions; *t*-tests compared continuous variables. Linear regression models assessed predictors of total and physician reimbursement, with results reported as regression coefficients (95% CI). Logistic regression models assessed the predictive value of surgical setting on adverse events, with results reported as odds ratios (95% CI).

## 3. Results

**Figure 1:**
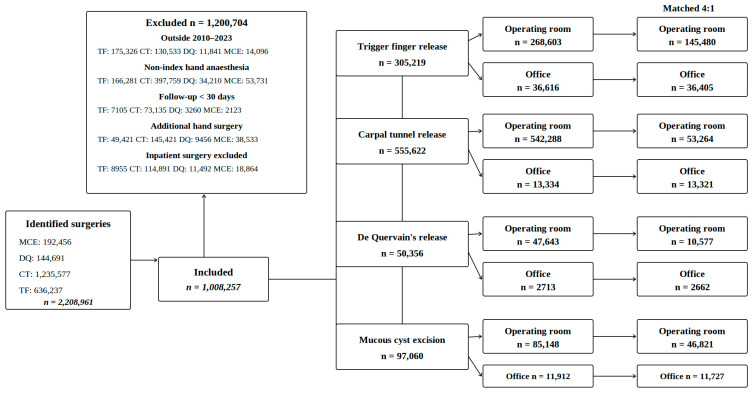
Flowcharts showing designation of each surgical cohort.

**Figure 2:**
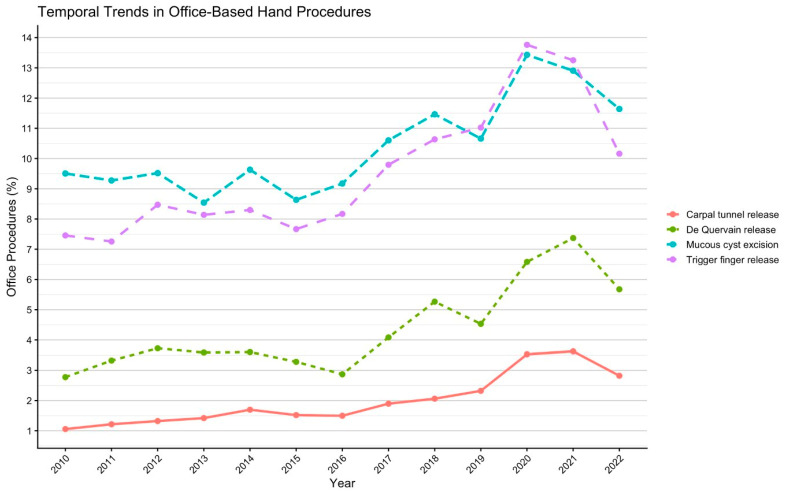
Annual percentage of total surgeries (office plus operating room) taking place in the office from 2010 to 2022.

Patient Selection and Volume Trends. There were 1,229,491 total patients: trigger finger release (381,206), carpal tunnel release (670,513), De Quervain’s release (61,848), and mucous cyst excision (115,924). Office-based surgeries comprised 36,616 (9.6%) trigger finger releases, 13,334 (2.0%) carpal tunnel releases, 2713 (4.4%) De Quervain’s releases, and 11,912 (10.3%) mucous cyst excisions. From 2010 to 2022, the annual proportion of office surgeries increased for all cohorts (Table 1; Figure 1 and Figure 2).

Patient Demographics. In unmatched cohorts, statistically significant differences existed between OR and office patients for most characteristics (Table 2). Following 1:4 matching by age, sex, ECI score, and region, there were no significant differences in these variables. Significant differences in payor mix persisted across all cohorts after matching, with OR patients having a higher proportion of Medicare coverage (Table 3).

Reimbursement. Office-based surgery had significantly lower total reimbursement for all surgical cohorts and insurance types: commercial −34% to −43%, Medicaid −37% to −48%, Medicare −30% to −37% (Table 4). Office-based surgery also had lower physician reimbursement for cohorts with commercial insurance (−3% to −9%) and Medicare (−5% to −13%). Physician reimbursement was not significantly different by surgical setting for Medicaid patients in any cohort. Linear regression confirmed office-based surgery as an independent predictor of reduced total reimbursement (coefficients −$661 to −$1029; all *p* < 0.001) (Table 5) and physician reimbursement for trigger finger, carpal tunnel, and De Quervain’s release (coefficients −$19 to −$53; *p* < 0.001), but not mucous cyst excision (Table 6).

Postoperative Outcomes. Office-based surgery was associated with significantly fewer filled narcotic prescriptions for all cohorts (ORs 0.47–0.61, *p* < 0.001). Lower 90-day ED visit rates were observed for trigger finger release (4.2% vs. 5.3%, OR 0.79, *p* < 0.001), carpal tunnel release (5.0% vs. 6.8%, OR 0.72, *p* < 0.001), De Quervain’s release (4.9% vs. 6.1%, OR 0.78, *p* = 0.012), and mucous cyst excision (3.5% vs. 4.2%, OR 0.83, *p* = 0.001). There were no significant differences in 90-day SSI rates across cohorts (Table 7).

## 4. Discussion

The current study examines trends and outcomes in wide-awake OR- and office-based hand surgery in the United States from 2010 to 2022, finding that office-based surgery became more common, was associated with reduced financial burden and narcotics prescriptions, and had low rates of adverse events. The current study expands on prior single-center studies demonstrating reduced cost and similar postoperative outcomes for office-based surgery, providing population-level generalizability across common hand procedures [10,11]. We also build on our prior work which examined individual wide-awake procedures, including carpal tunnel release [12,13], Dupuytren fasciectomy [14], and flexor tendon repair [15], by simultaneously analyzing four common wide-awake procedures in national cohorts.

Office surgery has many purported benefits, but it remains uncommon. From 2010 to 2022, the proportion of office surgery increased substantially for all four surgical cohorts, indicating that office surgery is becoming increasingly popular as its benefits are recognized. Importantly, in 2020, the COVID-19 pandemic likely played a role in increasing the proportion of surgeries in the office. While the pandemic had wide-reaching effects on the healthcare economy and significantly decreased the overall volume of elective surgery, these changes may have reduced surgeries in the hospital setting to a greater extent than those in the office, thus inflating the proportion of office-based surgeries [17]. Even in 2022, two years after the peak of the COVID-19 pandemic, the proportion of wide-awake surgeries performed in the office remained small. Potential barriers to transitioning more hand surgeries to the office include patient perception, surgeon preference, and differences in reimbursement [18]. Facility fees from ambulatory surgical centers are another important consideration. In the US, 90% of ambulatory surgery centers (ASCs) are partially owned by physicians and 65% are solely physician-owned, which may represent a significant obstruction to many hand surgeons shifting surgeries to the office setting [19].

The total cost of wide-awake hand surgery was significantly lower for office-based surgery in all four surgical cohorts and across insurance types, which aligns with other studies showing cost reductions in the office and suggests that office-based surgery is associated with greatly reduced financial burden on the healthcare system [9,10,11]. The total cost of wide-awake hand surgery in the OR was up to $483 more expensive than office-based surgery. This difference multiplied by the vast number of these common hand surgeries represents a massive financial burden on the healthcare system, and payors should consider incentivizing surgeons to shift simple, low-risk surgeries to the office.

The current study found that physician reimbursement was lower in the office than in the OR, a finding that was contrary to the initial hypothesis that reimbursement for office-based surgery would be greater as an incentive to reduce costs for payors. When considering reimbursement, it is important to note that some surgeons have reported that they can perform twice as many cases per day in the office compared to the OR [20]. Although increasing procedural efficiency depends heavily on each practice’s clinical environment and workflow, many surgeons and practices could benefit financially from office-based surgery despite reduced payments. Because the difference in reimbursement is small relative to the total payment, performing just one more procedure in the office per day could eliminate the deficit. Additional factors such as ownership of ambulatory surgical centers must also be considered in the evaluation of financial benefit.

Notably, insurance coverage and geographic region were associated with both total and physician reimbursement. Commercial insurance reimbursed at the greatest rates, and Medicare reimbursed at the lowest rates, which aligns with recent evidence showing reductions in Medicare payments [21,22]. Regional variation in reimbursement for hand surgery has been previously documented [23,24,25]. In the current study, total reimbursement was generally greatest in the Midwest, followed by the West. Interestingly, however, the pattern of regional variation changes for physician reimbursement. For example, residence in the South was associated with significantly greater total reimbursement for carpal tunnel release but had no significant association with physician reimbursement for that procedure, suggesting that much of the regional variation in total reimbursement is driven by facility and ancillary costs rather than physician payments. While these findings demonstrate that significant regional variation exists in total and physician reimbursement for common hand surgeries, the current study does not adequately control for the many potential underlying causes. Further investigations tailored to this question may be warranted.

For all surgical cohorts, the percentage of patients who filled narcotic prescriptions was significantly lower following office-based surgery, a finding concordant with our prior report of reduced perioperative opioid prescriptions for office-based wide-awake carpal tunnel release specifically [13]. Other prior studies have shown that wide-awake surgery is not associated with increased narcotic utilization, but the current study corroborates our previous findings which demonstrated that surgical setting itself may be associated with reduced narcotic utilization for wide-awake surgery [26,27]. Differences in narcotic utilization could be due to a number of factors, including surgeon prescribing habits and patient perceptions and inclinations. Patients may have reduced expectations for pain due to reassurances of the simplicity of surgery during patient education, and the perioperative milieu of the OR may prime patients for anxiety, which could affect the perception of pain. Additionally, patients who are inclined to undergo office-based surgery may be less anxious or fearful than those who prefer surgery in the OR, which could reduce their desire to fill narcotics prescriptions and reduce the likelihood that their surgeon prescribes narcotics proactively. While there was a significant difference in narcotic usage between our groups, it is important to note that the forces underlying changes in prescribing patterns are complex and can be attributed to various social, legal, and political influences. The current study was not designed to adequately control for many of the relevant variables. This finding is important to acknowledge and should encourage further investigation into the impact of surgical setting on opioid prescriptions but should be interpreted cautiously in isolation.

Patients with office-based surgery had similar or reduced 90-day rates of postoperative acute care utilization and similar rates of surgical site infection compared to those with surgery in the OR. This aligns with prior studies of adverse events [9]. The association of office-based surgery with reduced ED utilization is likely multifactorial and may involve psychosocial patient factors as discussed previously. Although the current study was unable to evaluate patient-reported outcome measures, prior studies have demonstrated positive findings for office-based surgery [28,29].

These findings should also be considered alongside established clinical guidance that directly addresses the utilization and outcomes of hand surgery performed outside the traditional operating room. In 2022, the British Society for Surgery of the Hand (BSSH), in conjunction with the National Health Service Getting It Right First Time (GIRFT) programme and with endorsement from the British Association of Plastic, Reconstructive and Aesthetic Surgeons, issued formal guidance identifying hand surgeries such as open carpal tunnel release, trigger finger release, Dupuytren fasciectomy, flexor tendon repair and mucous cyst excision as appropriate for office or procedure room settings. This decision was based on systematic evidence review and clinical consensus [30]. That guidance describes two supporting case series: an elective ambulatory unit in the United Kingdom, separately published with a 1.36% infection rate for carpal tunnel decompression and patient satisfaction of 9.8 of 10 [31], and a Canadian series reporting a 0.39% infection rate across more than 11,000 procedures [30]. Separately, the 2024 American Academy of Orthopaedic Surgeons/American Society for Surgery of the Hand Clinical Practice Guideline for the Management of Carpal Tunnel Syndrome likewise recognizes that carpal tunnel release may be safely performed in the office setting with outcomes comparable to the operating room. However, in the U.S. this recommendation is currently graded as being supported by limited evidence [32]. The nationwide increase in office-based utilization and the favorable safety profile demonstrated in the current study help address this evidence gap and support the appropriateness criteria set forth in these guidelines.

The current study has several limitations. Typical of an insurance claims database study, the data relies on the accuracy of administrative coding. Given the large sample sizes, small inaccuracies in coding should have minimal effects on the results. Additionally, the database does not directly capture certain technical elements that define the WALANT technique such as the use of epinephrine or the absence of a tourniquet. Wide-awake cases were instead identified indirectly by excluding CPT codes for nerve blocks, sedation, monitored anesthesia care, and general anesthesia. As a result, the identified cohort may include some procedures performed under local anesthesia that do not strictly meet the classic WALANT definition, and this should be considered when interpreting the results. The study also has no method for assessing patient-reported outcome measures (PROMs), patient satisfaction scores, subjective pain, muscle strength, range of motion, limb function, long-term recurrence, or return-to-work data, which are important in comparing the surgical settings. Because these core clinical indicators of hand surgery outcome were not captured, the clinical value of office-based surgery cannot be fully demonstrated by this study, which may limit its use in supporting large-scale clinical adoption. While the current findings support the economic efficiency and short-term safety of office-based surgery, they cannot directly assess patient experience, functional recovery, or patient-perceived value, and the results should not be extrapolated to overall healthcare value without this context. Future studies incorporating PROMs, patient satisfaction measures, subjective pain scores, muscle strength and range of motion assessments, and long-term recurrence data would be valuable to further validate the broader clinical implications of these findings. The current study controlled for geographic differences by matching patients based on United States Census Bureau definitions of the four primary geographic regions. However, these are broadly defined regions of nonuniform size that include both rural and urban areas. This approach, therefore, reduces confounding but is an imperfect control. Notably, the study uses the amount paid by payors rather than the amount charged. The authors view this as a strength of the study, as it allows cost to be represented by the amount of money that was exchanged rather than amounts charged to payors, which are then reimbursed to varying degrees based on unknown agreements.

## 5. Conclusions

Office-based wide-awake hand surgery is increasing in prevalence and is associated with substantially lower total costs, reduced narcotic prescriptions, and similar or lower rates of adverse events compared to OR-based surgery. These findings support the economic efficiency and short-term safety of office-based surgery for eligible patients. However, patient-reported outcomes, satisfaction, pain, and functional data were not available in this database. Future studies incorporating these measures are needed to more fully characterize patient-centered value. With this context, hand surgeons may consider performing more procedures in the office when appropriate.

## Figures and Tables

**Table 1:** Trends in office-based surgery, 2010 vs. 2022.

Procedure	2010 *n*	Office %	2022 *n*	Office %	*p*-Value
Trigger finger release	29,585	7.5%	36,689	10.2%	<0.001
Carpal tunnel release	59,789	1.1%	60,027	2.8%	<0.001
De Quervain’s release	4794	2.8%	5832	5.7%	<0.001
Mucous cyst excision	10,151	9.5%	9149	11.6%	<0.001

*n* = total annual surgical volume. Office % = proportion of surgeries performed in the office. *p*-values compare 2010 to 2022 proportions via chi-squared test.

**Table 2:** Demographics of unmatched cohorts.

	Trigger Finger			Carpal Tunnel			De Quervain’s			Mucous Cyst		
	OR	Office	*p*	OR	Office	*p*	OR	Office	*p*	OR	Office	*p*
N	344,590	36,616		657,179	13,334		59,135	2713		104,012	11,912	
Age, mean (SD)	62 (11)	63 (11)	<0.001	59 (14)	58 (14)	<0.001	54 (14)	54 (14)	0.064	59 (13)	61 (12)	<0.001
Female, *n* (%)	224,235 (65)	23,020 (63)	<0.001	408,374 (62)	8326 (62)	0.769	48,434 (82)	2134 (79)	<0.001	70,069 (67)	8082 (68)	0.293
Male, *n* (%)	120,354 (35)	13,596 (37)	—	248,804 (38)	5008 (38)	—	10,701 (18)	579 (21)	—	33,943 (33)	3830 (32)	—
ECI, mean (SD)	4.0 (3.4)	4.0 (3.4)	0.385	3.7 (3.3)	3.8 (3.3)	0.001	3.3 (3.2)	3.6 (3.3)	<0.001	2.9 (2.9)	2.9 (2.9)	0.330
Northeast, *n* (%)	69,567 (20)	4418 (12)	<0.001	127,303 (19)	1581 (12)	<0.001	12,356 (21)	361 (13)	<0.001	23,604 (23)	1644 (14)	<0.001
South, *n* (%)	126,737 (37)	11,011 (30)	—	238,282 (36)	3783 (28)	—	24,064 (41)	958 (35)	—	36,012 (35)	4132 (35)	—
West, *n* (%)	48,567 (14)	8409 (23)	—	86,949 (13)	2744 (21)	—	7280 (12)	439 (16)	—	14,046 (14)	2442 (21)	—
Midwest, *n* (%)	98,223 (29)	12,595 (34)	—	201,521 (31)	5158 (39)	—	15,152 (26)	939 (35)	—	29,926 (29)	3620 (30)	—
Medicare, *n* (%)	89,185 (26)	8846 (24)	<0.001	156,011 (24)	2695 (20)	<0.001	9427 (16)	408 (15)	0.001	19,949 (19)	2389 (20)	<0.001
Medicaid, *n* (%)	13,239 (4)	1284 (4)	—	40,117 (6)	975 (7)	—	3364 (6)	188 (7)	—	3358 (3)	311 (3)	—
Commercial, *n* (%)	233,928 (68)	25,693 (70)	—	445,837 (68)	9369 (70)	—	44,747 (76)	2058 (76)	—	78,055 (75)	8927 (75)	—

ECI = Elixhauser Comorbidity Index. *p*-values from *t*-test (continuous) or chi-squared test (categorical). — indicates *p*-value not separately reported for subcategories.

**Table 3:** Demographics of matched cohorts.

	Trigger Finger			Carpal Tunnel			De Quervain’s			Mucous Cyst		
	OR	Office	*p*	OR	Office	*p*	OR	Office	*p*	OR	Office	*p*
N	145,480	36,405		53,264	13,321		10,577	2662		46,821	11,727	
Age, mean (SD)	63 (10)	63 (10)	0.594	58 (14)	58 (14)	0.979	54 (14)	54 (14)	0.944	61 (12)	61 (12)	0.837
Female, *n* (%)	91,630 (63)	22,924 (63)	0.962	33,261 (62)	8319 (62)	1.000	8407 (79)	2111 (79)	0.856	31,887 (68)	7983 (68)	0.959
Male, *n* (%)	53,850 (37)	13,481 (37)	—	20,003 (38)	5002 (38)	—	2170 (21)	551 (21)	—	14,934 (32)	3744 (32)	—
ECI, mean (SD)	3.9 (3.3)	3.9 (3.4)	0.738	3.8 (3.3)	3.8 (3.3)	0.893	3.4 (3.1)	3.5 (3.1)	0.632	2.8 (2.7)	2.8 (2.7)	0.674
Northeast, *n* (%)	17,640 (12)	4411 (12)	0.986	6318 (12)	1580 (12)	1.000	1399 (13)	352 (13)	0.901	6548 (14)	1639 (14)	0.920
South, *n* (%)	43,990 (30)	11,001 (30)	—	15,119 (28)	3780 (28)	—	3804 (36)	952 (36)	—	16,357 (35)	4093 (35)	—
West, *n* (%)	33,110 (23)	8288 (23)	—	10,961 (21)	2742 (21)	—	1671 (16)	423 (16)	—	9387 (20)	2353 (20)	—
Midwest, *n* (%)	50,180 (34)	12,557 (34)	—	20,614 (39)	5155 (39)	—	3669 (35)	923 (35)	—	14,391 (31)	3601 (31)	—
Medicare, *n* (%)	39,716 (27)	8775 (24)	<0.001	11,569 (22)	2690 (20)	<0.001	1769 (17)	399 (15)	0.003	10,305 (22)	2326 (20)	<0.001
Medicaid, *n* (%)	5253 (4)	1268 (3)	—	3564 (7)	974 (7)	—	590 (6)	183 (7)	—	1240 (3)	303 (3)	—
Commercial, *n* (%)	96,998 (67)	25,569 (70)	—	36,855 (69)	9362 (70)	—	7944 (75)	2022 (76)	—	34,059 (73)	8818 (75)	—

Patients were matched 1:4 (office:OR) by age, sex, ECI score, and U.S. Census region using exact matching. — indicates *p*-value not separately reported for subcategories.

**Table 4:** Total and physician reimbursement, matched cohorts.

	Trigger Finger			Carpal Tunnel			De Quervain’s			Mucous Cyst		
	OR	Office	*p*	OR	Office	*p*	OR	Office	*p*	OR	Office	*p*
Total reimbursement, mean (SD)												
Commercial	$966 (1421)	$548 (589)	<0.001	$1108 (1738)	$734 (1048)	<0.001	$1039 (1452)	$598 (553)	<0.001	$1154 (1542)	$671 (886)	<0.001
Medicaid	$768 (1206)	$406 (494)	<0.001	$837 (1228)	$526 (699)	<0.001	$824 (1606)	$502 (750)	<0.001	$941 (1759)	$494 (542)	<0.001
Medicare	$480 (779)	$302 (316)	<0.001	$544 (1207)	$381 (560)	<0.001	$495 (818)	$331 (397)	<0.001	$569 (1010)	$362 (565)	<0.001
Physician reimbursement, mean (SD)												
Commercial	$714 (719)	$671 (623)	<0.001	$796 (641)	$747 (609)	<0.001	$644 (696)	$585 (420)	<0.001	$559 (432)	$545 (369)	0.003
Medicaid	$441 (453)	$417 (402)	0.071	$547 (520)	$513 (438)	0.050	$416 (435)	$378 (321)	0.238	$373 (293)	$384 (285)	0.577
Medicare	$382 (352)	$353 (308)	<0.001	$408 (329)	$372 (295)	<0.001	$340 (249)	$296 (231)	0.001	$294 (211)	$280 (202)	0.002

Reimbursement values are mean amounts paid by payors (SD) on the day of surgery. *p*-values from *t*-tests comparing OR vs. office.

**Table 5:** Predictors of total reimbursement (linear regression).

	Trigger Finger Coef. (95% CI)	*p*	Carpal Tunnel Coef. (95% CI)	*p*	De Quervain’s Coef. (95% CI)	*p*	Mucous Cyst Coef. (95% CI)	*p*
Office surgery	−$852 (−871, −833)	<0.001	−$1029 (−1058, −1000)	<0.001	−$732 (−783, −681)	<0.001	−$661 (−682, −641)	<0.001
Age (per year)	−$6 (−7, −5)	<0.001	−$9 (−10, −8)	<0.001	−$1 (−3, 0)	0.094	−$2 (−3, −1)	<0.001
Female sex	−$66 (−82, −50)	<0.001	−$49 (−74, −25)	<0.001	−$4 (−55, 46)	0.865	$0 (−18, 18)	0.966
ECI score	−$7 (−10, −5)	<0.001	$0 (−3, 4)	0.800	−$20 (−27, −13)	<0.001	−$17 (−20, −14)	<0.001
South (ref: Northeast)	−$122 (−148, −96)	<0.001	$248 (208, 289)	<0.001	−$64 (−130, 2)	0.059	−$46 (−72, −19)	<0.001
West (ref: Northeast)	$185 (157, 212)	<0.001	$353 (311, 395)	<0.001	$158 (81, 234)	<0.001	$152 (123, 181)	<0.001
Midwest (ref: Northeast)	$205 (179, 231)	<0.001	$651 (613, 690)	<0.001	$347 (280, 413)	<0.001	$288 (262, 315)	<0.001
Medicaid (ref: Medicare)	$150 (104, 196)	<0.001	$121 (66, 177)	<0.001	$86 (−20, 191)	0.111	$50 (−7, 106)	0.087
Commercial (ref: Medicare)	$728 (709, 747)	<0.001	$782 (750, 813)	<0.001	$603 (542, 663)	<0.001	$570 (548, 593)	<0.001

Coefficients represent the expected change in total day-of-surgery reimbursement for a one-unit increase in the predictor, holding other variables constant.

**Table 6:** Predictors of physician reimbursement (linear regression).

	Trigger Finger Coef. (95% CI)	*p*	Carpal Tunnel Coef. (95% CI)	*p*	De Quervain’s Coef. (95% CI)	*p*	Mucous Cyst Coef. (95% CI)	*p*
Office surgery	−$19 (−26, −11)	<0.001	−$43 (−54, −32)	<0.001	−$53 (−78, −27)	<0.001	−$8 (−16, 1)	0.067
Age (per year)	−$2 (−3, −2)	<0.001	−$3 (−4, −3)	<0.001	−$1 (−2, 0)	0.171	$0 (−1, 0)	0.112
Female sex	−$21 (−28, −14)	<0.001	−$6 (−17, 4)	0.240	$6 (−22, 34)	0.688	$9 (1, 17)	0.020
ECI score	−$15 (−16, −14)	<0.001	−$28 (−30, −26)	<0.001	−$20 (−24, −16)	<0.001	−$16 (−18, −15)	<0.001
South (ref: Northeast)	−$100 (−111, −88)	<0.001	−$1 (−19, 16)	0.889	−$153 (−190, −116)	<0.001	−$76 (−88, −65)	<0.001
West (ref: Northeast)	$44 (32, 56)	<0.001	$103 (84, 121)	<0.001	$34 (−9, 76)	0.124	$15 (3, 28)	0.018
Midwest (ref: Northeast)	$26 (15, 37)	<0.001	$149 (132, 166)	<0.001	$19 (−18, 56)	0.303	$20 (8, 32)	<0.001
Medicaid (ref: Medicare)	$43 (23, 63)	<0.001	$67 (42, 91)	<0.001	$49 (−9, 108)	0.098	$69 (44, 94)	<0.001
Commercial (ref: Medicare)	$294 (286, 302)	<0.001	$306 (293, 320)	<0.001	$263 (230, 296)	<0.001	$237 (228, 247)	<0.001

Coefficients represent the expected change in physician reimbursement for a one-unit increase in the predictor, holding other variables constant.

**Table 7:** Postoperative outcomes, matched cohorts.

	TF OR *n* (%)	TF Office *n* (%)	OR (95% CI)	*p*	CT OR *n* (%)	CT Office *n* (%)	OR (95% CI)	*p*	DQ OR *n* (%)	DQ Office *n* (%)	OR (95% CI)	*p*	MCE OR *n* (%)	MCE Office *n* (%)	OR (95% CI)	*p*
Narcotics prescription (90-day)	74,178 (51.0%)	12,484 (34.3%)	0.50 (0.49, 0.51)	<0.001	31,063 (58.3%)	6134 (46.0%)	0.61 (0.59, 0.63)	<0.001	6175 (58.4%)	1061 (39.9%)	0.47 (0.43, 0.52)	<0.001	23,066 (49.3%)	3744 (31.9%)	0.48 (0.46, 0.50)	<0.001
Emergency dept. visit (90-day)	7701 (5.3%)	1536 (4.2%)	0.79 (0.74, 0.83)	<0.001	3622 (6.8%)	668 (5.0%)	0.72 (0.66, 0.78)	<0.001	641 (6.1%)	131 (4.9%)	0.78 (0.64, 0.95)	0.012	1968 (4.2%)	416 (3.5%)	0.83 (0.75, 0.93)	0.001
Surgical site infection (90-day)	534 (0.4%)	118 (0.3%)	0.88 (0.72, 1.07)	0.216	179 (0.3%)	37 (0.3%)	0.82 (0.57, 1.16)	0.275	28 (0.3%)	*	—	—	144 (0.3%)	41 (0.3%)	1.14 (0.79, 1.59)	0.472

Odds ratios (OR) compare office to operating room (reference). TF = trigger finger; CT = carpal tunnel; DQ = De Quervain’s; MCE = mucous cyst excision. * PearlDiver suppresses cell counts below 11.

## Data Availability

Restrictions apply to the availability of these data. Data were obtained from PearlDiver (M170Ortho dataset) and are available from the authors with the permission of PearlDiver Technologies.

## Abbreviations

WALANT	Wide-Awake Local Anesthesia No Tourniquet
OR	Operating Room
ECI	Elixhauser Comorbidity Index
ED	Emergency Department
SSI	Surgical Site Infection
TF	Trigger Finger
CT	Carpal Tunnel
DQ	De Quervain’s
MCE	Mucous Cyst Excision
ICD	International Classification of Diseases
CPT	Current Procedural Terminology
